# Assessment of Clinical Signs of Liver Cirrhosis Using T1 Mapping on Gd-EOB-DTPA-Enhanced 3T MRI

**DOI:** 10.1371/journal.pone.0085658

**Published:** 2013-12-31

**Authors:** Michael Haimerl, Niklas Verloh, Florian Zeman, Claudia Fellner, René Müller-Wille, Andreas G. Schreyer, Christian Stroszczynski, Philipp Wiggermann

**Affiliations:** 1 Department of Radiology, University Hospital Regensburg, Regensburg, Germany; 2 Center for Clinical Trials, University Hospital Regensburg, Regensburg, Germany; University of Navarra School of Medicine and Center for Applied Medical Research (CIMA), Spain

## Abstract

**Objectives:**

To assess the differences between normal and cirrhotic livers by means of T1 mapping of liver parenchyma on gadoxetic acid (Gd-EOB-DTPA)-enhanced 3 Tesla (3T) MR imaging (MRI).

**Methods:**

162 patients with normal (n = 96) and cirrhotic livers (n = 66; Child-Pugh class A, n = 30; B, n = 28; C, n = 8) underwent Gd-EOB-DTPA-enhanced 3T MRI. To obtain T1 maps, two TurboFLASH sequences (TI = 400 ms and 1000 ms) before and 20 min after Gd-EOB-DTPA administration were acquired. T1 relaxation times of the liver and the reduction rate between pre- and post-contrast enhancement images were measured.

**Results:**

The T1 relaxation times for Gd-EOB-DTPA-enhanced MRI showed significant differences between patients with normal liver function and patients with Child-Pugh class A, B, and C (p < 0.001). The T1 relaxation times showed a constant significant increase from Child-Pugh class A up to class C (Child-Pugh class A, 335 ms ± 80 ms; B, 431 ms ± 75 ms; C, 557 ms ± 99 ms; Child-Pugh A to B, p < 0.001; Child-Pugh A to C, p < 0.001; Child-Pugh B to C, p < 0.001) and a constant decrease of the reduction rate of T1 relaxation times (Child-Pugh class A, 57.1% ± 8.8%; B, 44.3% ± 10.2%, C, 29.9% ± 6.9%; Child-Pugh A to B, p < 0.001; Child-Pugh A to C,p < 0.001; Child-Pugh B to C, p < 0.001).

**Conclusion:**

Gd-EOB-DTPA-enhanced T1 mapping of the liver parenchyma may present a useful method for determining severity of liver cirrhosis.

## Introduction

Cirrhosis, the final stage of chronic diffuse liver disease, is histologically defined as the presence of regenerative nodules surrounded by fibrous bands in response to chronic liver injury, resulting in the distortion of the hepatic vasculature and consecutive portal hypertension [1]. Numerous antifibrotic agents are currently developed that may delay progression to decompensated cirrhosis or even reverse cirrhosis; however, progress has been impeded by the low number of methods available for measuring the progression or reversal of fibrosis [2].

The presence of hepatitis-related fibrosis is an indication for antiviral treatment, and the presence of cirrhosis is an indication for the specific monitoring of complications related to portal hypertension and the increased risk of hepatocellular carcinoma. Thus, the early detection of hepatic fibrosis and cirrhosis has important clinical implications. In clinical routine, determination of the severity of liver injury and the requirement of antiviral therapy is often based on the histology of the liver parenchyma [3,4].

Biopsy is thus considered the gold standard for the diagnosing and staging of cirrhosis. The risk of disease progression can be monitored by the sequential histological grading of inflammation as well as by the staging of fibrosis [2]. However, liver biopsy is poorly accepted by patients and has a risk of various complications, such as hemorrhage and infection. In addition, liver biopsies may be prone to sampling errors and inter-observer variability due to subjective morphological evaluation [5,6]. Therefore, non-invasive quantitative markers or diagnostic tests are required to assess the presence and severity of liver cirrhosis. Several non-invasive diagnostic methods for fibrosis or cirrhosis have been evaluated. These methods mainly include either clinical signs, ultrasonographic signs, or biochemical blood parameters [7]. The recent advent of ultrasound-based imaging of tissue elasticity, such as acoustic radiation force impulse (ARFI) elastography, represents a non-invasive way to quantify liver fibrosis. However, ARFI measurements have only limited intra- and interoperator reproducibility [8].

Gadolinium ethoxybenzyl diethylenetriaminepentaacetic acid (Gd-EOB-DTPA) is a hepatocyte-specific contrast agent for MRI of the liver (Bayer Schering Pharma, Berlin, Germany). The additional value of Gd-EOB-DTPA compared with other gadolinium-based contrast agents is the selective uptake of the contrast medium by functioning hepatocytes. In turn, this uptake can be used to acquire MR images during the hepatobiliary phase (HP), which occurs approximately 15 to 20 min after the injection of the contrast medium [9]. Therefore, this agent can be used for dynamic bolus phase MRI as well as for liver-specific (hepatocyte-phase) MRI within the same examination within a reasonable time frame, thus combining the properties of an extracellular and a hepatocyte-specific contrast agent [10]. As a result of the organic anion-transporting polypeptide-1 (OATP1) dependent intracellular uptake of Gd-EOB-DTPA and its T1-shortening effect, functioning areas of the liver parenchyma exhibit T1 shortening. Furthermore, livers with normal hepatocyte function show increased signal intensity during the HP, indicating that the use of Gd-EOB-DTPA has the potential to directly measure liver function [11,12].

Assessment of hepatic functional reserve plays a crucial role in selecting therapeutic approaches in patients with liver tumors as well as in preventing hepatic failure after treatment, particularly in patients with chronic liver disease. The Child-Pugh Score is commonly used to determine the prognosis of chronic liver disease, such as liver cirrhosis. 

Thus, the purpose of our study was to evaluate the usefulness of Gd-EOB-DTPA-enhanced MRI in diagnosing hepatic cirrhosis, to distinguish between different types of cirrhosis, and to evaluate liver function using the T1 relaxation time of liver parenchyma.

## Materials and Methods

### Patients

Between April 2012 and April 2013, 216 consecutive patients with known liver cirrhosis, suspected chronic liver disease or suspicious focal hepatic lesions detected during previous ultrasonography or computed tomography examinations underwent liver MRI including T1 mapping in our department. 54 patients were excluded from the analysis, because they had either received a liver transplant (n = 7) or previous local treatment for liver disease (n = 36), or their images had severe motion artifacts due to a poor breath-holding technique (n = 11).

Finally, 162 patients were enrolled into our study: 96 patients with normal liver function (31 women and 65 men; mean age, 59.2 years) and 66 patients with liver cirrhosis (15 women and 51 men; mean age, 60.4 years). The non-cirrhotic liver group was chosen among patients with normal liver laboratory values and without direct or indirect signs (e.g. splenomegaly) of liver disease in ultrasound. Diagnosis of liver cirrhosis was based on laboratory values, imaging findings and clinical findings according to the Child-Pugh Score depending on the degree of ascites (1 point, none; 2 points, mild; 3 points, moderate to severe), serum bilirubin (mg/dl, 1 point, < 2; 2 points, 2 - 3; 3 points, > 3), serum albumin (g/dl, 1 point, < 3.5; 2 points, 2.8 - 3.5; 3 points, < 2.8), prothrombin time (INR, 1 point, < 1.7; 2 points, 1.71- 2.3; 3 points, > 2.3), and encephalopathy (1 point, none; 2 points, Grade I-II; 3 points, Grade III- IV). 30 patients of the 66 patients with liver cirrhosis were classified with Child-Pugh A (LCA, 5 - 6 points), 28 patients with Child-Pugh B (LCB, 7 - 9 points), and 8 patients with Child-Pugh C (LCC, 10 - 15 points). Patient characteristics are shown in Table 1.

**Table 1:** Patient characteristics.

Group	All (n=162)	NLF (n = 96)	LCA (n = 30)	LCB (n = 28)	LCC(n = 8)
Age	59.7 ± 12.2	59.2 ± 12.8	62.1 ± 11.0	59.8 ± 12.4	56.1 ± 8.3
Gender					
male	116 (72%)	65 (68%)	22 (73%)	25 (89%)	4 (50%)
female	46 (28%)	31 (32%)	8 (27%)	3 (11%)	4 (50%)
Height	1.73 ± 0.09	1.72 ± 0,1	1.73 ± 0.08	1.75 ± 0.08	1.72 ± 0.1
Weight	82.1 ± 17.9	80.0 ± 16.0	85.9 ± 19.3	87.3 ± 19.7	75.9 ± 23.4
BMI	27.4 ± 5.5	26.9 ± 5.0	28.6 ± 6.5	28.6 ± 6.5	25.7 ± 7.8

Values indicate mean ± standard deviation.

NLF: normal liver function; LCA: liver cirrhosis Child-Pugh A; LCB: liver cirrhosis Child-Pugh B; LCC: liver cirrhosis Child-Pugh C; BMI: body mass index

### Ethics statement

This prospective study was approved by the Institutional Review Board of the University of Regensburg. Written informed consent was obtained from all patients.

### MR imaging protocol

All MRI examinations were conducted with a clinical 3T whole body system (Magnetom Skyra, Siemens Healthcare, Erlangen, Germany). For signal reception, a combination of body and spine array coil elements (3T; 18-channel body matrix coil, 24-channel spine matrix coil) was used in all examinations.

At first, coronal respiratory-triggered single-shot T2-weighted turbo spin-echo imaging was performed followed by breath-hold transverse fast-spoiled gradient-echo images, i.e. T1-weighted in-phase and T1-weighted out-of-phase imaging. Then, a transverse T1-weighted volume interpolated breath hold examination (VIBE) sequence with fat suppression was acquired before contrast media injection and during dynamic phases in arterial phase (20 s), late arterial phase (50 s) and portal venous phase (80 s). All patients received a body weight adapted dose of Gd-EOB-DTPA (0.025mmol/kg body weight) administered via bolus injection with a flow rate of 1 mL/s, flushed with 20 mL NaCl. Also axial respiratory-triggered single-shot T2-weighted turbo spin-echo images, respiratory-triggered BLADE sequence with fat suppression and diffusion weighted images were acquired. Finally, transverse and coronal breath-hold T1-weighted VIBE sequence was repeated in hepatobiliary phase 20 min after contrast media administration.

In addition to the routine MRI protocol, two TurboFLASH sequences (inversion time (TI), 400 ms, 1000 ms; repetition time (TR), 4000 ms; echo time (TE), 1.16 ms; flip angle 8°; slice thickness, 6 mm; FOV 400 mm x 400 mm; matrix size, 192 x 192; acquisition time 16s) were acquired to obtain T1 maps of the porta hepatis in a single breath-hold before and 20 min after Gd-EOB-DTPA administration.

### Image analysis

The T1 maps of the liver were generated with the evaluation tool for calculating T1 relaxation times (Siemens Healthcare, Erlangen, Germany). 

In T1 mapping images before and after the administration of the contrast medium, regions of interest (ROIs) were located with reasonable care in the right lobe (2 ROIs) and in the left lobe (1 ROI). We avoided focal hepatic lesions (e.g. hepatocellular carcinoma, hemangioma, cysts, etc.), imaging artifacts, and major branches of the portal or hepatic veins. Each ROI was a circle (size of the ROI ranged between 1.0 cm^2^ and 3.5 cm^2^) chosen as large as possible. Mean T1 values for the three ROIs were considered as the representative T1 value for the liver. ROIs of identical size and shape were placed at the same imaging sequence in T1 maps before and after Gd-EOB-DTPA administration. The reduction rate in T1 values between pre- and post-Gd-EOB-DTPA enhancement was calculated as follows:

$$R\text{eduction rate of T1 values = }\left[\frac{T1_{pre}-T1_{post}}{T1_{pre}}\right]\times 100\;(\%)$$

T1pre is the T1 relaxation time before Gd-EOB-DTPA administration and T1post the T1 relaxation time 20 min after Gd-EOB-DTPA administration. T1 maps were color-coded using a visualization tool of the open source OsiriX imaging software.

### Statistical analysis

Data are expressed as mean ± standard deviation (SD). A one-way analysis of variance (ANOVA) was used to analyze differences between patients with normal liver function and patients with liver cirrhosis. Post hoc pair-wise comparisons were made with the Tukey procedure. ROC analyses were done to differentiate between patient groups, and the optimal cut-off was estimated according to the Youden Index. Estimates for the area under the curve (AUC) as well as true classification rates are reported. All tests were two-sided and values of p < 0.05 indicated a significant difference. All statistical analyses were done with IBM SPSS Statistics (version 20, Chicago, IL) and R 2.14. 

## Results

Including the results of all patients, the T1 relaxation times of non-enhanced MRI (774.2 ms ± 123 ms) and Gd-EOB-DTPA-enhanced MRI (322.3 ms ± 113 ms) were significantly different (p < 0.001): the mean reduction rate was 58.3% ± 13%.

**Figure 1:**
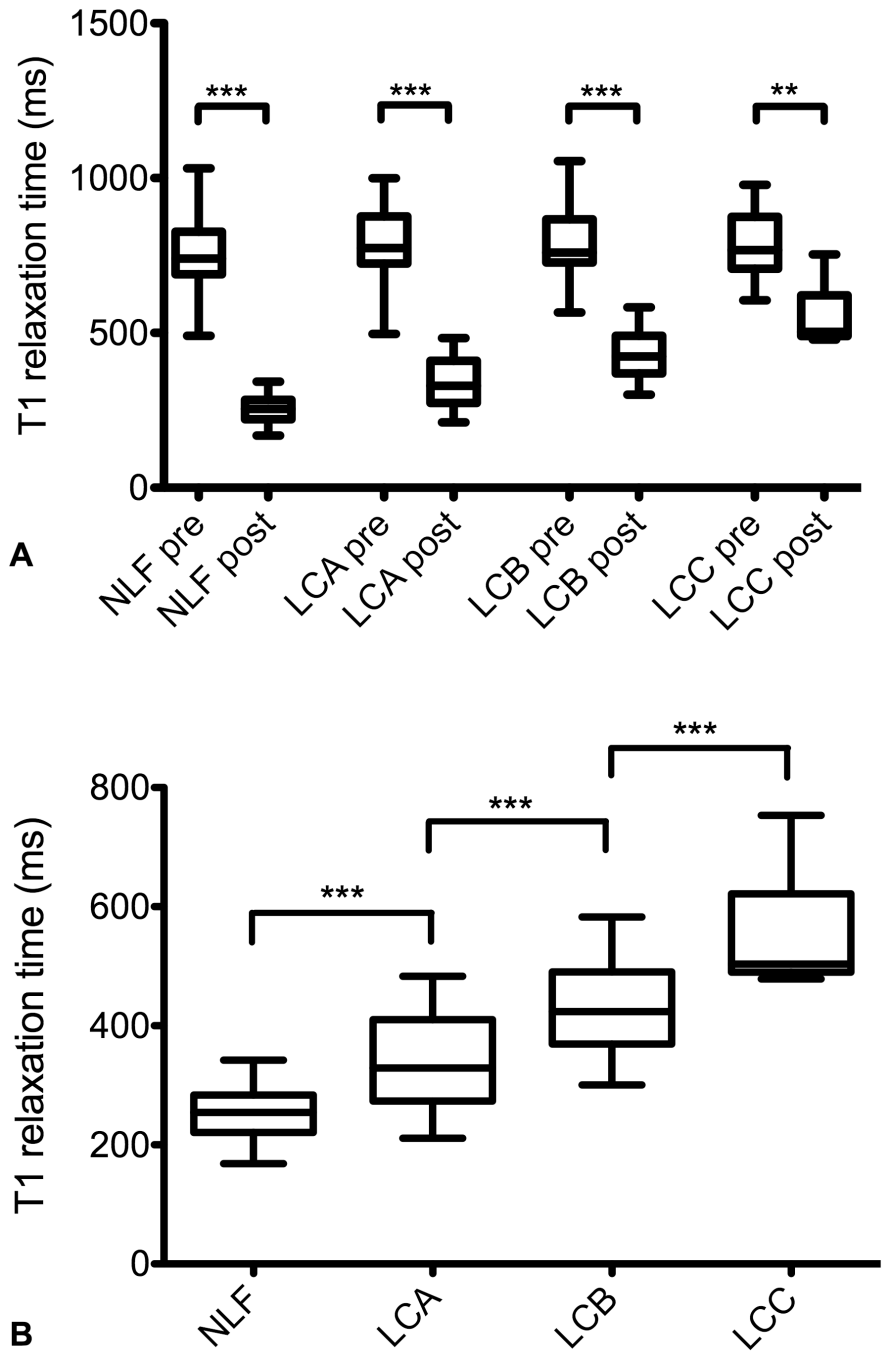
Pre- and post-contrast T1 relaxation times. (A) Boxplots indicating T1 relaxation times before (pre) and after (post) Gd-EOB-DTPA administration in patients with normal liver function and in patients with liver cirrhosis Child-Pugh A, Child-Pugh B, and Child-Pugh C. After contrast medium administration, T1 relaxation times were significantly reduced in each group. (B) Boxplots indicating T1 relaxation times after Gd-EOB-DTPA administration in patients with normal liver function and in patients with liver cirrhosis Child-Pugh A, Child-Pugh B, and Child-Pugh C. T1 relaxation times increased significantly with increased progression of liver cirrhosis. NLF: normal liver function; LCA: liver cirrhosis Child-Pugh A; LCB: liver cirrhosis Child-Pugh B; LCC: liver cirrhosis Child-Pugh C. Data given as mean T1 reduction rate ± standard deviation. Tukey’s adjustment was used to generate Boxplots, and the Wilcoxon-Test was used to compare groups. ***p ≤ 0.001, ** ≤ 0.01.

The pre-contrast T1 relaxation time of the liver parenchyma showed no significant difference between patients with normal liver function (NLF) and patients with LCA, LCB, and LCC (NFL to LCA, p = 0.90, 95% CI (-84.67 to 49.25); NFL to LCB, p = 0.90, 95% CI (-86.88 to 50.64); NLF to LCC; p = 0.97, 95% CI (-138.64 to 96.98)). After the administration of Gd-EOB-DTPA, T1 relaxation times decreased significantly between patients with normal liver function and patients classified according to the Child-Pugh system (p < 0.001), (Figure 1A, Table 2). 

**Table 2:** T1 relaxation times of the liver in non-enhanced and Gd-EOB-DTPA-enhanced MRI.

	**non-enhanced (ms)**	**Gd-EOB-DTPA- enhanced (ms)**
NLF	767 ± 129	267 ± 76
LCA	784 ± 110	335 ± 80
LCB	784 ± 114	431 ± 75
LCC	788 ± 123	557 ± 99

Values indicate mean ± standard deviation.

NLF: normal liver function; LCA: liver cirrhosis Child-Pugh A; LCB: liver cirrhosis Child-Pugh B; LCC: liver cirrhosis Child-Pugh C

A comparison between patients with normal liver function and patients with liver cirrhosis stratified by the Child-Pugh classification showed that T1 relaxation times were prolonged in case of cirrhosis: The T1 relaxation times showed a constant significant increase from the NLF group to the LCC group (NFL to LCA, p < 0.001, 95% CI (-111.40 to -26.71); NFL to LCB, p < 0.001, 95% CI (-208.59 to -121.62); NLF to LCC; p < 0.001, 95% CI (-365.34 to -216.34)), (Figure 1B).

**Figure 2:**
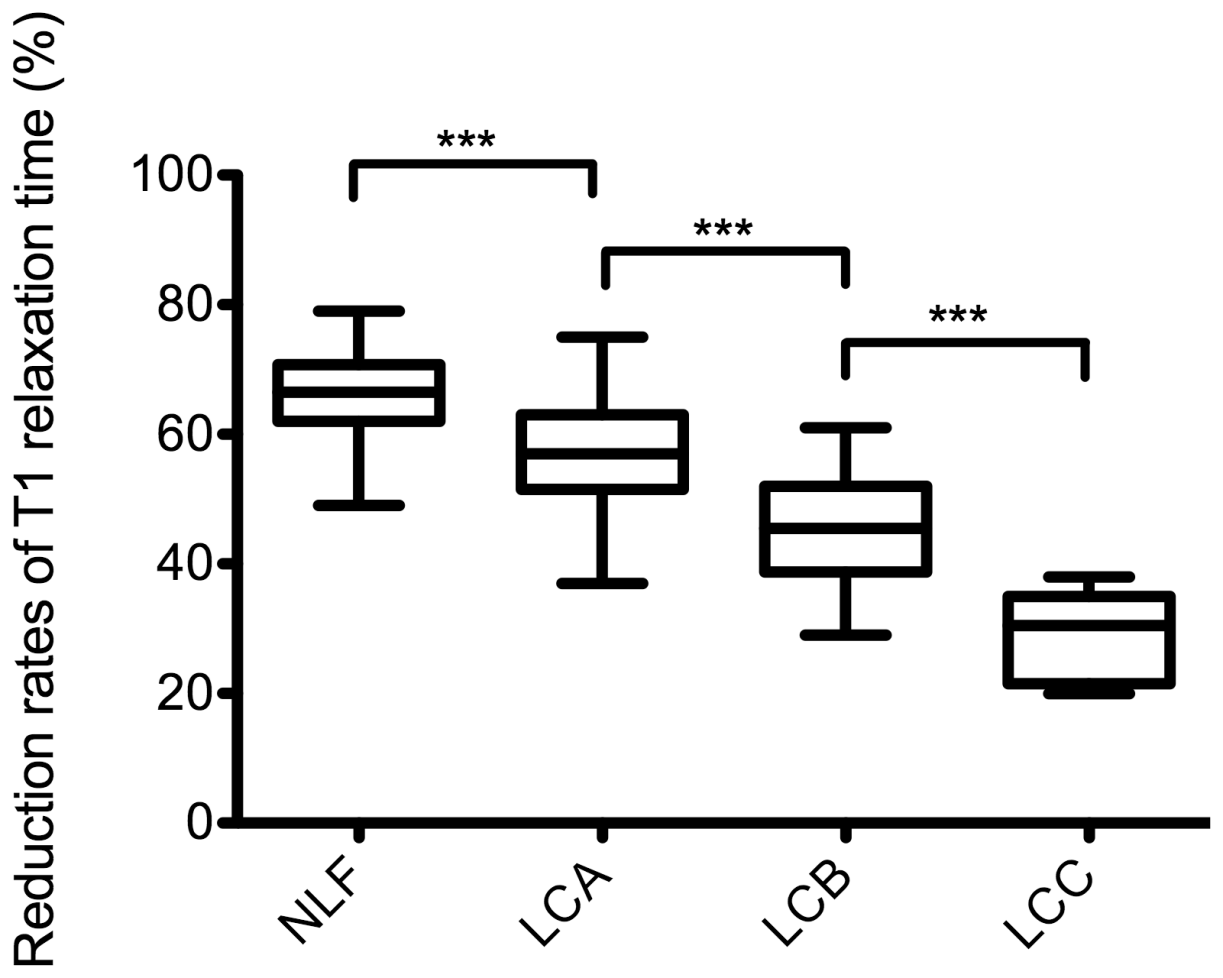
Reduction rates of T1 relaxation times. Boxplots of reduction rates of T1 relaxation times of the liver in patients with normal liver function and in patients with liver cirrhosis classified as Child-Pugh A, Child-Pugh B, and Child-Pugh C. Reduction rates were significantly reduced with increasing progression of liver cirrhosis. NLF: normal liver function; LCA: liver cirrhosis Child-Pugh A; LCB: liver cirrhosis Child-Pugh B; LCC: liver cirrhosis Child-Pugh C. Data given as mean T1 reduction rate ± standard deviation. Tukey’s adjustment was used to generate Boxplots, and the Wilcoxon-Test was used to compare groups. ***p ≤ 0.001.

20 min after contrast medium administration, reduction rates of T1 relaxation times were significantly lower for patients with liver cirrhosis compared to patients with NLF (p ≤ 0.001). A constant significant decrease of the reduction rate of T1 relaxation times could be shown (NFL to LCA, p < 0.001, 95% CI (0.04 to 0.12); NFL to LCB, p < 0.001, 95% CI (0.16 to 0.25); NLF to LCC; p < 0.001, 95% CI (0.28 to 0.44)), (Figure 2, Table 3).

**Table 3:** Reduction rates of T1 relaxation times of the liver in non-enhanced and Gd-EOB-DTPA-enhanced MRI.

	Reduction rates of T1 relaxation time (%)
NLF	65.1 ± 7.1
LCA	57.1 ± 8.8
LCB	44.3 ± 10.2
LCC	29.9 ± 6.9

Values indicate mean ± standard deviation.

NLF: normal liver function; LCA: liver cirrhosis Child-Pugh A; LCB: liver cirrhosis Child-Pugh B; LCC: liver cirrhosis Child-Pugh C

**Figure 3:**
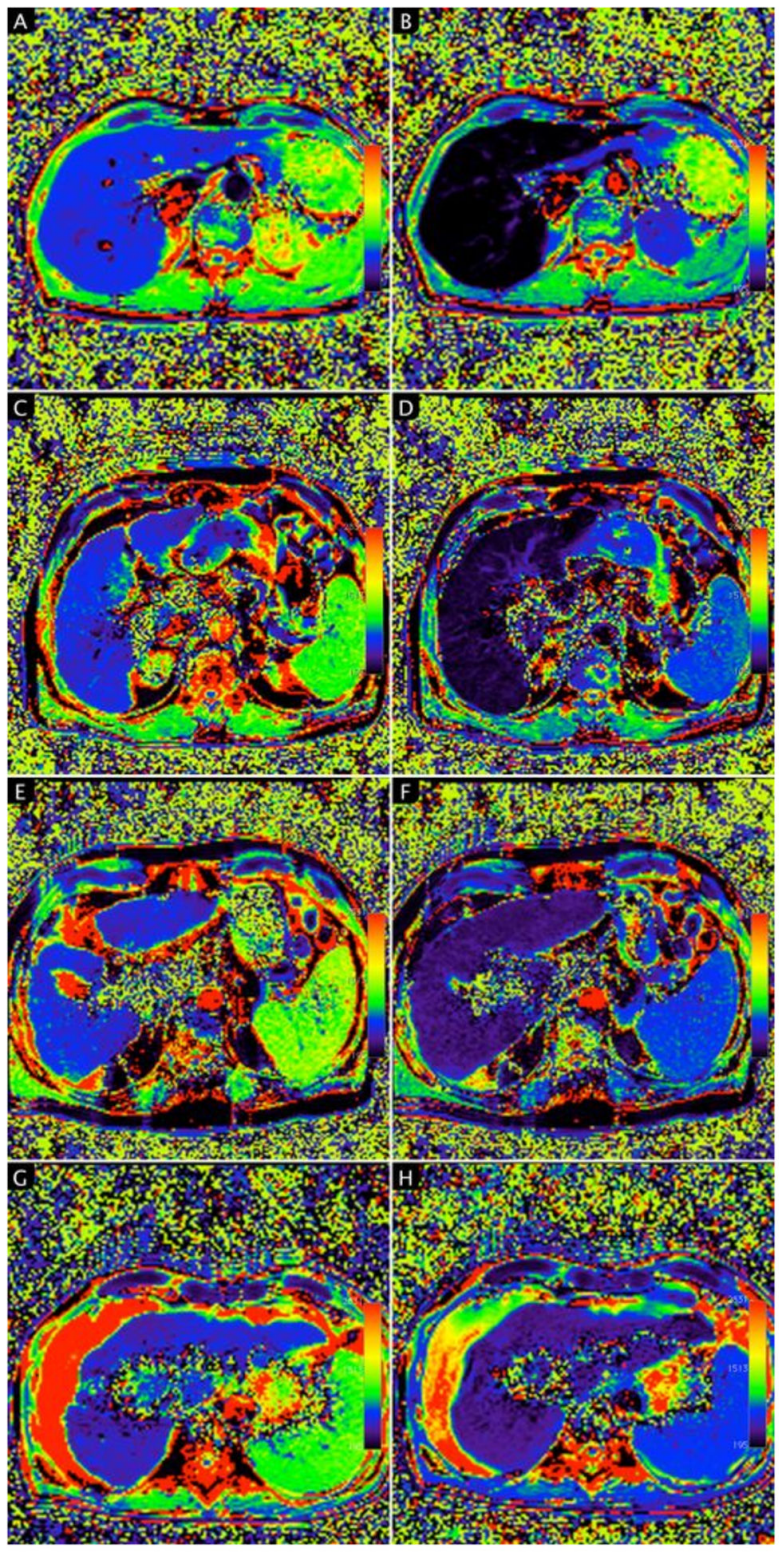
T1 relaxation times of the liver on T1 mapping color-coded images. A - H, T1 mapping images calculated from 2 TurboFlash sequences (TI = 400 ms and 1000 ms) were obtained before (A, C, E, G) and 20 min after Gd-EOB-DTPA administration (B, D, F, H) in a patient with normal liver function (A, B) and patients with liver cirrhosis Child-Pugh A (C, D), Child-Pugh B (E, F), and Child-Pugh C (G, H). The mean T1 relaxation times of liver parenchyma were as follows: 755.6 ms (A), 211.9 ms (B), 717.1 ms (C), 316.0 ms (D), 760.5 ms (E), 434.0 ms (F), 705.7 ms (G) and 482.4 ms (H). The reduction rate of T1 relaxation times in patients with normal liver function and patients with liver cirrhosis Child-Pugh A, Child-Pugh B, and Child-Pugh C were 72%, 56%, 43%, and 32%, respectively. In patients with normal liver function, T1 relaxation times on T1 mapping color-coded images of the liver on post-contrast MRI (B) showed a markedly darker color distribution of the liver parenchyma than that on the pre-contrast mapping image (A), indicating a strong Gd-EOB-DTPA-induced shortening of T1 relaxation time 20 min after contrast medium administration. With increased progression of liver cirrhosis, the color distribution in the liver parenchyma could be visually confirmed to show less change on post-contrast T1- mapping images.

Color-coded T1 relaxation times on T1 mapping images from the NLF group to the LCC group on pre- and post-contrast MRI, respectively, are shown in Figure 3.

An ROC analysis was done with regard to cut-off values and area under the curve (AUC) values for differentiating patients with NLF from patients with liver cirrhosis as well as for differentiating different grades of cirrhosis based on the reduction rates. The cut-off value of T1 relaxation times to distinguish the NLF group from the liver cirrhosis groups LCA, LCB, and LCC were 419.8 ms, 389.1 ms, and 326.3 ms, respectively. The cut-off value to differentiate LCB from LCA and LCC was 329.5 ms and 325.5 ms. The cut-off values regarding the reduction rate of T1 relaxation times to differentiate the NLF group from the liver cirrhosis groups LCA, LCB, and LCC were 63.5%, 59.1%, and 40.9%, respectively. The cut-off values to differentiate LCB from LCA and LCC were 41.2% and 38.1%. Various cut-off values of T1 relaxation times and reduction rates, each with corresponding AUC values and true classification rates are shown in Table 4 and Table 5.

**Table 4:** ROC analysis indicating various cut-off values and diagnostic performance to differentiate patients with NLF from patients with liver cirrhosis LCA, LCB, and LCC: T1 relaxation times after Gd-EOB-DTPA administration.

	Cut-offs of T1 relaxation time (ms)	AUC (true classification rates)
NLF - LCA	419.8	0.627 (80%, 43%)
NLF - LCB	389.2	0.834 (91%, 75%)
NLF - LCC	326.3	0.996 (98%, 100%)
LCA - LCB	329.5	0.971 (90%, 100%)
LCB - LCC	325.5	0.835 (64%, 100%)

AUC: area under the receiver operating characteristic curve; NLF: normal liver function; LCA: liver cirrhosis Child-Pugh A; LCB: liver cirrhosis Child-Pugh B; LCC: liver cirrhosis Child-Pugh C

**Table 5:** ROC analysis indicating various cut-off values and diagnostic performance to differentiate patients with NLF from patients with liver cirrhosis LCA, LCB, and LCC: reduction rate of T1 relaxation between pre- and post-contrast times.

	Cut-offs of reduction rate (%)	AUC (true classification rates)
NLF - LCA	63.5	0.773 ( 67%, 80%)
NLF - LCB	59.1	0.962 ( 84%, 96%)
NLF - LCC	40.9	1.000 (100%, 100%)
LCA - LBB	41.2	0.996 ( 97%, 100%)
LCB - LCC	38.1	0.888 ( 79%, 100%)

AUC: area under the receiver operating characteristic curve; NLF: normal liver function; LCA: liver cirrhosis Child-Pugh A; LCB: liver cirrhosis Child-Pugh B; LCC: liver cirrhosis Child-Pugh C

## Discussion

Gd-EOB-DTPA is a liver-specific MRI contrast agent with combined dynamic perfusion and hepatocyte selective properties [13]. After intravenous injection, gadoxetate disodium is gradually taken up by hepatocytes. Approximately 50% of the agent is eventually excreted via the biliary pathway, whereas the percentage of the contrast medium not eliminated by the hepatobiliary system is cleared by glomerular filtration in the kidneys. Peak liver signal intensity has been observed during the hepatobiliary phase 20 min after contrast medium injection, followed by a plateau-like enhancement lasting for about 2 h [9,14]. The hepatocyte-specific uptake of Gd-EOB-DTPA is thought to be mediated by active membrane transport systems, such as OATP1, located at the sinusoidal membrane, and the multidrug resistance protein 2 (MRP2), located at the canalicular membrane of hepatocytes [15]. There is some evidence that Gd-EOB-DTPA-enhanced MRI may assess liver function.

In cirrhotic livers, hepatocyte function is impaired compared to that in non-cirrhotic livers. Cirrhotic liver parenchyma has shown reduced signal intensity in HB relative to non-cirrhotic liver parenchyma with a correlation between hepatic parenchymal enhancement and the Child-Pugh Score. However, measurements of signal intensities describe relative values, are easily affected by variable parameters, and involve high standard deviations indicating huge variances [12,16,17].

Gd-EOB-DTPA is a contrast medium with T1-shortening effects [18]. Therefore, we generated T1 maps indicating the absolute values of T1 relaxation times that did not considerably vary at various points of measurement and thus led to low values of standard deviation. 

After administration of Gd-EOB-DTPA, patients with normal liver function had the lowest values of T1 relaxation times (267 ms). These values showed a constant significant increase of T1 relaxation times from patients with normal liver function up to patients with Child-Pugh class C (LCA, 335 ms; LCB, 431 ms; LCC, 557 ms). However, shortening of T1 relaxation times after contrast medium administration in non-cirrhotic livers and prolonged post-contrast T1 relaxation times in cirrhotic livers may be modified by pre-contrast T1 relaxation times. To evaluate the actual intracellular uptake of Gd-EOB-DTPA, we assessed the relative change in T1 relaxation times defined as the reduction rate, which was 65.1% in non-cirrhotic patients. This percentage was further decreased with increased progression of liver cirrhosis (LCA, 57.1%; LCB, 44.3%, LCC, 29.9%). The reduced Gd-EOB-DTPA uptake may be either due to a decreased number of normal hepatocytes according to characteristic morphological changes in cirrhotic liver tissue or due to a decreased Gd-EOB-DTPA uptake by hepatocytes, attributed to diminished OATP1 activity, resulting in lower Gd-EOB-DTPA intracellular hepatocyte uptake [19]. In animal models, the expression of organic anion-transporting proteins has been reported to decrease in hepatitis and cirrhosis and to up-regulate MRP2 activity [20,21].

However, it is still uncertain whether morphologically advanced cirrhosis would impair the uptake of Gd-EOB-DTPA and, in turn, reduce liver parenchymal enhancement after Gd-EOB-DTPA administration because of the expected impairment of hepatocyte function or the decreased number of hepatocytes. In this context, it has been reported that decreased hepatic enhancement in patients with impaired hepatocyte function may impede the detection of focal liver lesions because of the reduced contrast between the lesion and the surrounding liver parenchyma [22]. Therefore, the detection of focal liver lesions in patients with impaired liver function, such as severe cirrhosis, also represents a challenge in daily clinical routine.

Because the intracellular uptake of Gd-EOB-DTPA decreases with impaired liver function, measurement of T1 relaxation times in Gd-EOB-DTPA-enhanced images may be a new, non-invasive technique to quantify the actual function of hepatocytes.

In line with the controversial findings in the current literature [23,24], we did not find any significant differences in T1 relaxation times between patients with normal liver function and patients with liver cirrhosis in non-enhanced images (NLF: 767 ms ± 129 ms; LCA: 784 ms ± 110 ms; LCB: 784 ms ± 114 ms; LCC, 788 ms ± 123 ms). Liver cirrhosis involves increased deposition of manganese, copper, iron, and collagen ─ paramagnetic macromolecules that induce T1-shortening effects [25,26]. However, characteristics of T1 relaxation times in healthy and cirrhotic patients has been discussed controversially in the literature: Contrary to our findings, Thomson et al. indicated that tissue remodeling in liver cirrhosis may be reflected in prolonged T1 relaxation times [27], a conclusion that has been supported by other recent studies [17,28]. These findings have been validated in some recent animal studies that showed an increase in T1 relaxation times in CCL4-induced liver fibrosis. However, this increase is most likely caused by pathophysiological processes of induced liver fibrogenesis characterized by edema, inflammation, and synthesis of the extracellular matrix [29,30]. At this early stage of fibrous formation, the tissue is often edematous because new vessels have leaky interendothelial junctions, allowing the passage of proteins and red blood cells into the extravascular space [31]. This process results in augmented hepatic water content, hypercellularity, and an increase in the ratio of free to bound water with consecutive prolonged T1 relaxation times [32,33]. However, increases of T1 relaxation times could also be observed in a bile-duct ligation model, which is another common method to induce liver fibrosis, resulting in minimal inflammation and limited edema. Such increases are most likely due to an increase in water-containing small biliary ducts [34]. 

Advanced stages of cirrhosis show increased extracellular constituents, mostly collagen, and a decreased number of active fibroblasts and new vessels, resulting in lower total water contents and consecutively decreased T1 relaxation times. In the clinical setting, liver cirrhosis is mostly an expression of chronic liver disease and is not commonly combined with acute inflammation. Kim et al. have recently reported a significant shortened T1 value in patients with liver cirrhosis measured by means of an unenhanced 3T system [35]. These controversial findings indicate that non-enhanced MRI is unlikely to be an appropriate method for detecting and staging liver fibrosis. 

Our study supports the conclusion that measuring T1 relaxation times by means of Gd-EOB-DTPA-enhanced MRI may be incorporated into the clinical routine as a screening test of liver imaging to detect silent disease and to define the stage of existing disease or liver function in pre- or postoperative assessments, while extending the acquisition time of a liver MRI protocol by only 32 s. 

Our study has several limitations. First, the trial was a single-center study with a limited patient population. Second, our study included various types of cirrhosis, such as cirrhosis induced by alcohol, cirrhosis based on longtime viral hepatitis, or a combination of both. Future studies should use a homogenous patient population because the etiology of cirrhosis has an impact on parenchymal changes and may thus influence the resulting T1 maps. 

Third, some evidence exists that clinical Child-Pugh scores are related to histopathological fibrosis, and Child-Pugh classification has been proven to correctly assess disease stages in clinical practice. However, results of T1 mapping needs to be evaluated in comparison to histopathological results to prove that disease progression into higher Child-Pugh classes also reflects progressive parenchymal changes.

Finally, we compared the hepatic intracellular uptake of Gd-EOB-DTPA only with the Child-Pugh classification but did not evaluate other liver function tests, such as ICG test. Therefore, further studies are required in this respect.

In conclusion, T1 mapping by means of non-enhanced and Gd-EOB-DTPA-enhanced MRI may provide suitable and robust parameters for detecting and characterizing liver cirrhosis at an early stage. Additionally, this method may be useful for monitoring disease progression and has the potential to become a novel index of global and remnant liver function. 
